# "It is her responsibility": partner involvement in prevention of mother to child transmission of HIV programmes, northern Tanzania

**DOI:** 10.1186/1758-2652-14-21

**Published:** 2011-04-26

**Authors:** Eli Fjeld Falnes, Karen Marie Moland, Thorkild Tylleskär, Marina Manuela de Paoli, Sia E Msuya, Ingunn MS Engebretsen

**Affiliations:** 1 Centre for International Health, University of Bergen, Bergen, Norway; 2 Bergen University College, Bergen, Norway; 3 Fafo Institute for Applied International Studies, Oslo, Norway; 4 Department of Community Health, Tumaini University, Kilimanjaro Christian Medical College, Moshi, Tanzania

## Abstract

**Background:**

Partner involvement has been deemed fundamental in prevention of mother to child transmission (PMTCT) programmes, but is difficult to achieve. This study aimed to explore acceptability of the PMTCT programme components and to identify structural and cultural challenges to male involvement.

**Methods:**

The study was conducted during 2007-2008 in rural and urban areas of Moshi in the Kilimanjaro region of Tanzania. Mixed methods were used, and included focus group discussions with fathers and mothers, in-depth interviews with fathers, mothers and health personnel, and a survey of 426 mothers bringing their four-week-old infants for immunization at five reproductive and child health clinics.

**Results:**

Routine testing for HIV of women at the antenatal clinic was highly acceptable and appreciated by men, while other programme components, notably partner testing, condom use and the infant feeding recommendations, were met with continued resistance. Very few men joined their wives for testing and thus missed out on PMTCT counselling. The main barriers reported were that women did not have the authority to request their husbands to test for HIV and that the arena for testing, the antenatal clinic, was defined as a typical female domain where men were out of place.

**Conclusions:**

Deep-seated ideas about gender roles and hierarchy are major obstacles to male participation in the PMTCT programme. Empowering women remains a huge challenge. Empowering men to participate by creating a space within the PMTCT programme that is male friendly should be feasible and should be highly prioritized for the PMTCT programme to achieve its potential.

## Background

During the past decade, male involvement has been recognized as a priority area for the prevention of mother to child transmission (PMTCT) of HIV programmes [1]. The male partner plays a role in terms of a woman's risk of acquiring HIV [2] and in terms of her utilization of the PMTCT programme: for the mother to test for HIV [3-6], for the mother to return for the result [6], for the couple to use condoms [7,8], for the mother to receive medication [6,7,9] and for her to follow the infant feeding advice given [7,9-13].

In several studies, mainly from sub-Saharan Africa, the fear of a partner's negative reaction towards the mother testing for HIV and fear of disclosure of the test results [4,5,14-16] have been found to be barriers to HIV testing for pregnant women in the PMTCT programme. At the same time, many studies have shown that these negative attitudes ascribed to men are often exaggerated. Contrary to the anticipated fear, many men have been found to be quite supportive of their partners participating in the PMTCT programme [14-17]. Nevertheless, very few partners participate in antenatal HIV counselling and testing [7,9,18-20]. This is also the case in Tanzania, where the estimated prevalence of HIV in pregnant women attending antenatal care during 2007 was 8.2% [21].

Acknowledging the low male involvement in PMTCT programmes in the region, this study aimed at exploring the acceptability of the PMTCT programme components and to identify structural and cultural challenges to male involvement. More specifically, the study explored men's attitudes to the testing procedure and partner disclosure, condom use and infant feeding recommendations.

## Methods

**Figure 1:**
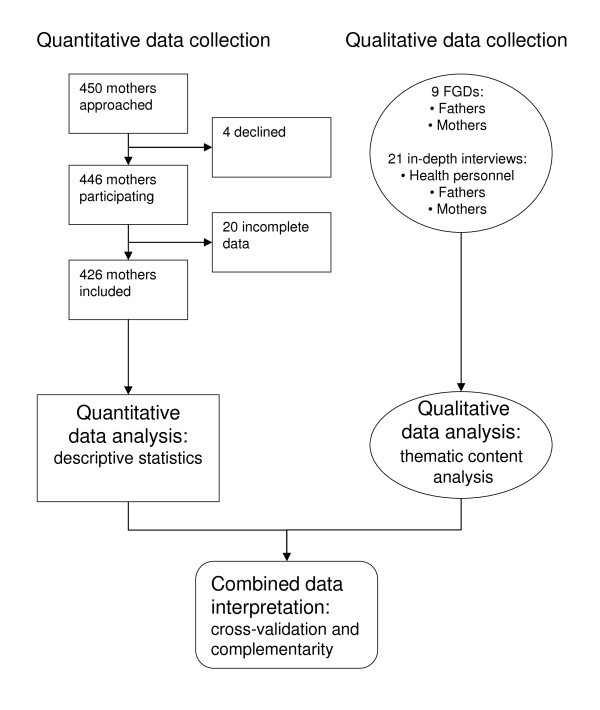
**Mixed methods: concurrent triangulation**.

This study forms part of a wider study on mothers' utilization of the PMTCT services and the methods employed have been described in detail elsewhere [22]. Briefly, mixed methods with a concurrent triangulation design were utilized [23] (Figure 1). A cross-sectional survey was conducted concurrently with qualitative in-depth interviews and focus group discussions (FGDs). In the study described in this article, the quantitative data served to complement the qualitative data obtained. The quantitative and qualitative data were analyzed separately and integrated during interpretation of the results.

### Study setting

The study was conducted from October 2007 to February 2008 at five reproductive and child health clinics in urban and rural areas of the Moshi District in the Kilimanjaro region in northern Tanzania. Moshi Town is the urban centre and the regional capital, with a population of 144,739 (2002 Census) [24]. The Chagga is the dominant ethnic group in terms of numbers, as well as political influence.

Because of shortage of land, labour migration has been extensive, and the HIV prevalence was high in the early phase of the epidemic [25,26]. The region hence was selected to pilot a PMTCT programme in 2000 and has now run PMTCT services for a decade. The Kilimanjaro region enjoys a reputation as one of the most modern regions in the country. The education level and utilization of health services is generally higher than in other regions in Tanzania [27-29].

Nevertheless, customary law still has a strong hold in rural areas and prohibits women from inheriting land and livestock [28]. Women's access to resources is mainly through men as fathers, husbands and sons. Women are accorded status based primarily on their reproductive capacities [27]. Breastfeeding is universal and 98.4% of children in this region have ever been breastfed [29]. A mixed feeding pattern, however, with early introduction of water, other fluids and porridge, in addition to breast milk, are common [30].

### The PMTCT programme

The national PMTCT programme in Tanzania was launched in five hospitals in 2000 as a pilot project [31]. The programme has thereafter expanded countrywide [32]. Studies carried out in the Kilimanjaro region before and during the pilot phase of the programme emphasized the importance of partner involvement [11,12,33]. The national PMTCT guidelines issued in 2004, which were the official ones during the time of the fieldwork, stated that all mother and child health facilities should initiate strategies to encourage pregnant women to attend the PMTCT programme together with their partners [34]. However, by the end of 2009, only 8% of male partners of pregnant women were aware of their HIV status [32].

The PMTCT programme in the region at the time of the study offered routine counselling and testing to all mothers when they came for their first antenatal visit [34]. Both pre- and post-test counselling was offered. During the post-test counselling, the mothers were asked to bring their partners to the clinic for testing. If the mother succeeded in bringing her partner to the clinic, couple counselling and testing was offered. The mother was informed about the importance of using condoms until both partners had tested negative.

Information given about infant feeding for HIV-infected mothers was in accordance with the 2001 guidelines from the World Health Organization (WHO) [35]. The mothers were offered three options: (a) exclusive breastfeeding for six months or early cessation at any time convenient to the individual woman's situation; (b) replacement feeding with commercial infant formula; and (c) replacement feeding with home-modified cow's milk.

Mother who tested positive were referred to care and treatment clinics for CD4 count and possible treatment. They were also informed about the importance of safe delivery at a clinic or hospital. Treatment and safe delivery, however, are not addressed in this study, as they were considered less controversial with regard to male involvement.

### Quantitative data

Five reproductive and child health clinics were purposefully selected to represent urban and rural areas of Moshi District. During the data collection period, each mother who attended one of the five clinics with her infant for the first dose of the diphtheria, pertussis, tetanus, hepatitis B (DPT-HB) and polio immunization was invited to participate in the study. The region has a coverage of 100% of this type of immunization [29]. Nursing staff at the clinics was informed about the purpose of the study and they explained it to each mother before enquiring about participation. In total, 450 mothers were approached; 446 (99.1%) agreed to participate. Of these, 20 were excluded from the study owing to incomplete data (Figure 1). More details on the survey have been described elsewhere [22].

Four female research assistants, including the main research assistant, conducted the interviews. The questionnaire was designed to collate information relating to socio-demographic characteristics; clinic attendance; place of birth; infant feeding practices; PMTCT practices at the clinic including counselling and testing for HIV; knowledge about PMTCT; and the relationship to a male partner. Information concerning HIV status was not collected.

Data were double entered into Epidata 3.1 software http://www.epidata.dk and analyzed using SPSS PASW. Descriptive statistics were used.

In addition, the PMTCT antenatal clinic register books for 2007 at four of the recruitment clinics were viewed. These books included records concerning the number of women and men tested for HIV at that antenatal clinic during that year.

### Qualitative data

The mothers and the fathers in the FGDs and in-depth interviews were recruited from various villages in urban and rural Moshi. The villages were within the catchment areas of the clinics in the quantitative study. Participants with children less than one year old were purposively selected, assuming that they had been offered or exposed to the PMTCT programme in the most recent pregnancy. The participants were recruited by the main research assistant, her acquaintances in the respective villages, and village leaders.

A semi-structured interview guide was prepared for each group of participants. Themes included in the FGD and in-depth interview guides for the fathers were their knowledge about MTCT and its prevention; attitudes towards the PMTCT programme; views concerning HIV testing at the antenatal clinics; perceived attitudes to HIV-infected wives; attitudes to condom use within marriage; attitudes to couple counselling and testing; involvement in infant feeding; and views on how to increase male involvement in the PMTCT programme.

Nine FGDs were conducted: five with fathers and four with mothers. The FGDs had between five and 12 participants. A few of the participants were couples. The FGDs were conducted outdoors or in private homes, churches or school buildings. The group interaction seemed good overall. The group processes in the FGDs may have facilitated discussion about sensitive topics as compared with one-to-one interviews [36]. Although the moderator was female, the flow in the discussion with the fathers did not seem to differ from the discussions with the mothers. The FGDs were moderated by a nurse working in a local HIV organization. She had training and experience in conducting FGDs. The discussions were conducted in Swahili.

Twenty-one in-depth interviews were conducted: five with fathers, five with mothers and 11 with health personnel, made up of five PMTCT counsellors working at the five reproductive and child health clinics included in the study, four counsellors working at voluntary counselling and testing (VCT) centres and two employees at a local HIV organization. The fathers and mothers were interviewed in their homes. Health personnel at the various clinics were interviewed at work. The in-depth interviews provided an opportunity to explore the personal perceptions and experiences of men and women related to the role of fathers in PMTCT. All in-depth interviews were carried out by the principal investigator (EFF). The interviews with the health personnel were conducted in English, while the interviews with mothers and fathers were conducted in Swahili using the main research assistant as an interpreter. She was fluent in English and Swahili.

Each FGD and in-depth interview lasted for between 45 and 90 minutes. They were recorded with the consent of the participants and subsequently transcribed verbatim. Interviews conducted in Swahili were then translated into English.

#### Qualitative data analysis

The qualitative data were analyzed by the principal investigator, in collaboration with the co-authors. A thematic content approach, guided by the Graneheim and Lundman framework, was utilized [37]. The material was systematically read through in order to identify the meaning units. A meaning unit was defined as a string of the text that expressed a single coherent thought, up to the point that the coherent thought changed. The meaning units were coded using a describing cue related to what the text bit concerned, e.g., testing for HIV. Codes concerning the same subject were grouped together into categories.

The interview guide was used as a point of departure for grouping information, deductively. During the analysis, new categories were developed inductively, e.g., from the category, "attitudes to testing", to the category, "female responsibility". The underlying meaning of the categories was formulated into a theme, e.g., "gender roles". Information obtained during the in-depth interviews and FGDs was analyzed and merged according to the codes and themes. Illustrative quotations were selected. Original data were reassessed by the principal investigator and one of the co-authors (IMSE) after analysis in order to detect any concepts or information that had been missed and to meet consensus of opinion between the analysts.

### Ethics

The study was approved by the National Institute for Medical Research Tanzania, the Tanzanian Commission for Science and Technology, the Kilimanjaro Christian Medical Centre Ethical Research Committee and the Regional Committees for Medical and Health Research Ethics for Region West, Norway. All participants provided individual informed consent.

## Results

A brief summary of the results is provided in Table 1.

**Table 1:** Summary of results by fathers and mothers concerning the acceptability of the PMTCT programme components

PMTCT components	Fathers	Mothers
**Acceptability of routine HIV testing in PMTCT programmes**	- purely beneficial	- purely beneficial
	- for the sake of the baby	- for the sake of the baby
	- 100% of those asked accepted testing of their wives	- 100% accepted testing
		
**Testing practice of male partners**	- positive attitude	- advantageous
	- few had tested	- difficult to ask partner to test
	- main barrier: asked by their wife to attend a female arena	- desirable if he could be invited by others
		
**Expectations and experiences related to disclosure of HIV status**	- a responsibility to support	- fear of partner's reactions
	- a few would treat her badly due to lack of trust	- responsibility to disclose
		- 95.9% had disclosed to partner
		
**Partners' attitude to condom use**	- associated with distrust	- important for prevention
	- unacceptable within the marriage	- main barrier: partner's reluctance
	- needed to be his decision	- difficult to ask partner to use condoms
		
**Role of partner in safe infant feeding**	- needed to be informed about wife's HIV status to accept uncustomary infant feeding	- necessary to disclose HIV status to partner to be able to follow infant feeding guidelines
		
**How to include partners effectively in the programme**	- other than the wife invite partner to test	- other than the wife invite partner to test
	- offer partner testing in arenas other than the antenatal clinic	- offer partner testing in arenas other than the antenatal clinic

### Quantitative sample characteristics

Almost half the mothers lived in a rural area, and 90.1% were married or cohabiting (Table 2). Nearly half the mothers (43.7%) were Catholic, and the most common ethnic group was Chagga (62.4%). Approximately half the mothers (44.8%) and 60.6% of the fathers had completed secondary or higher education. In 87.1% of the households, the father of the child was reported to be the head of the household.

**Table 2:** Socio-demographic characteristics of women attending reproductive and child health clinics for childhood immunizations, and their partners

Background factor	Total N = 426 (%)
Residence	
Rural	193 (45.3)
Urban	233 (54.7)
Mothers age, years	
<= 25	219 (51.4)
> 25	207 (48.6)
Number of children	
1	169 (39.7)
2	132 (31.0)
>=3	125 (29.3)
Marital status	
Married/cohabiting	384 (90.1)
Single/divorced/widow	42 (9.9)
Religion	
Catholic	186 (43.7)
Protestant	162 (38.0)
Muslim/other	78 (18.3)
Ethnicity	
Chagga	266 (62.4)
Pare/other	160 (37.6)
Education, mother	
0-7	235 (55.2)
>=8	191 (44.8)
Fathers age, years	
<=30	229 (53.8)
>30	197 (46.2)
Education, father	
0-7	168 (39.4)
>=8	258 (60.6)
Head of household	
Father of the child	371 (87.1)
Other	55 (12.9)

### The acceptability of HIV testing

Almost all the mothers (97.7%) had been offered HIV testing at the antenatal clinic (Table 3). The majority (78.6%) reported that they had asked their partners for permission to be tested; all partners had consented. All mothers who had been offered HIV testing consented to being tested, and 95.9% shared the test results with their partners.

**Table 3:** HIV testing and disclosure of mothers attending reproductive and child health clinics for childhood immunizations

Practice	N	n (%)
**Mother offered HIV test**	426	416 (97.7)
**Asked partner for permission to test**	416^a^	327 (78.6)
**Partner agreed for her to test**	327^b^	327 (100.0)
**Mother tested**	416^a^	416 (100.0)
**Shared the test results with partner**	416^a^	399 (95.9)
**Counsellor suggested testing of partner**	426	407 (95.5)
**Partner as primary confidant**	426	263 (61.7)

^a ^10 mothers were not offered a test

^b ^10 mothers were not offered a test and 89 mothers did not ask their partners for permission

#### For the sake of the baby

The qualitative findings indicated that the majority of the mothers had discussed HIV testing with their partners before arriving at the antenatal clinic. Routine clinical activities, including HIV testing for pregnant women, appeared to be highly valued and accepted among the fathers. Fathers stated that it was important to follow advice provided at the antenatal clinic for the sake of the baby:

I am very happy about this [women testing for HIV at the antenatal clinic]. It is good. It prevents transmission of the virus to the child and the mother gets to know her health. (Father FGD)

Most of the fathers expressed the view that it was unnecessary for their wives to ask for permission to be tested at the antenatal clinic as it was part of the routine antenatal care:

It is good for couples to share things at home but if it [HIV testing] is at the clinic, it is compulsory, so she does not need to talk to me. (Father FGD)

Discussions with the fathers revealed that it was far less acceptable for a woman to go for voluntary counselling and testing (VCT) than to receive routine antenatal clinical care. It was considered to mean either that she suspected him of being unfaithful or that she had been unfaithful herself:

It is acceptable for pregnant women to test according to the clinical advice without permission. But if she is not pregnant and she plans to go for testing, she needs to go with me, the husband. (Father FGD)

Hence, testing of the woman as a clinical routine in the antenatal clinic was in contrast to voluntary counselling and testing defined as beyond the choice of the couple. It was rather seen as a commitment on the part of the mother to know her HIV status and to take the necessary precautions to secure an HIV-negative child. The testing of the partner, however, was understood as a matter of choice and met a lot of resistance among the men.

#### "Women should not tell us men what to do"

Almost all women (95.5%) were encouraged by the nurse counsellors to bring their partners to the antenatal clinic for testing (Table 3). However, according to the PMTCT antenatal clinic registers at four of the clinics, only 3% of the partners were tested in 2007. The nurse counsellors working at the recruitment clinics stated that very few partners attended the antenatal clinic and even fewer were tested for HIV.

Fathers generally seemed to have a favourable view of HIV testing, and the majority knew that they were requested to undergo testing at the antenatal clinic during their spouses' pregnancies. Nevertheless, most admitted that they had not been tested there. Common explanations were a lack of time, not seeing the benefits of testing, and a perception that they would have the same result as their wife. However, in the course of the discussion, deep-seated ideas about gender roles emerged as a bigger challenge to partner testing.

In the PMTCT programme, access to the father is gained through the mother attending the antenatal clinic. It is therefore her responsibility to ask her partner to test at the antenatal clinic. However, several fathers stated that social norms inhibited them from attending:

Generally our women should not tell us men what to do, even though the advice comes from the doctor. Our tradition does not allow the women to lead their men. I may personally agree to test for HIV, but the majority would wonder why they [health personnel] told the woman to bring me. (Father FGD)

The mothers cited the same barriers as the fathers. Most of the mothers claimed that they had asked their partner to come to the clinic and be tested, but that they had experienced great difficulty trying to persuade them. Very few succeeded:

My husband refused HIV testing. I asked him and he said: why should I go for testing while I am HIV negative? I was not happy because I wanted to know his status. He just gave me the go ahead for the test but he did not want to be tested himself. (Mother in-depth interview)

In general, mothers did not feel empowered to request their partners to undergo a HIV test. Several mothers expressed the wish that partners be invited by others:

When we advise our men to test, they don't want to comply. (Mother FGD)

#### The antenatal clinic as a female arena

Furthermore, the organization of the PMTCT programme inhibited men from participating. Several fathers did not attend the antenatal clinic owing to fear of the reactions of other men and feeling uncomfortable about the idea of being the only man present. Furthermore, antenatal clinic activities were perceived by many fathers as outside their responsibility:

It is her responsibility to go to the clinic to check the health of the baby and there they test her. (Father FGD)

There was substantial agreement in the interviews carried out with the fathers and mothers about how to increase the number of men tested. Two issues were brought up as important. First, it was perceived as important for men to be seen by as few people as possible when going for testing. Second, it was perceived as important to avoid the feeling of being the only man present. Therefore, VCT centres were perceived as preferable to a crowded female-dominated antenatal clinic:

Many men are reluctant to come to the antenatal clinics. But they think VCT centres are easier because not many people see them. (Father FGD)

The fathers and mothers seemed to share their view that couple VCT is beneficial. There was a consensus regarding the advantages related to receiving the information together, to be tested together, and to get the results together:

Couple counselling and testing is a good idea because that way you learn about your health status together. (Father in-depth interview)

However, according to VCT counsellors, few couples came for couple VCT; husband and wife were usually tested separately. According to the counsellors, men feared disclosure of a potential HIV-positive test result:

Technically, men go to another centre for testing and after realizing that they are HIV negative, they encourage their wife to go for couple counselling because they know that their status is going to be negative. (VCT counsellor)

Hence, the major barriers to male testing that were revealed during the discussions were related to customary gender roles. First, a woman should not tell a man what to do, and second, the antenatal clinic was perceived to be a female arena not acceptable for a man to enter.

### The acceptability of partner disclosure

#### "I would tell my husband only"

The majority of the mothers (61.7%) who participated in the survey stated that their partners would be their primary confidents if they were infected with HIV (Table 3). Similarly, almost all the mothers in the FGDs and in-depth interviews reported that they would have chosen their partners as their primary confidants. The mothers expressed the view that their partners were nearest to them:

My husband would be the first to know so that we can go together for the testing to find out about him also. I would hide [the HIV status] from everyone else. I would tell it to my husband only. (Mother in-depth interview)

However, the mothers found it difficult to predict their partners' reactions to them if they were found to be HIV infected. Many believed that if a man did not trust his wife, she would be blamed for bringing HIV into the marriage and she would risk being beaten and thrown out of their home:

The problem if you share the result with him is that he may say that you are the one who infected him and his family might start hating you. (Mother FGD)

A few fathers admitted that they would divorce an HIV-infected wife. The main argument put forward was that she had got the infection through adultery and therefore it was their right to divorce her:

There will be misunderstanding in the family and I will blame her much for that behaviour [unfaithfulness]. I will chase her out. It will be very hard to stay with her because I will think she may infect me. (Father FGD)

However, it seemed that the importance of disclosing a HIV-positive test result outweighed the fear of the partner's potential reactions to it. The mothers expressed their responsibility to tell their partners if their test were positive so that they could be tested and receive treatment if necessary. There was, however, a difference found between the responses from the mothers in the in-depth interviews and in the FGDs. The mothers in the in-depth interviews generally expressed a more favourable view of their partners than the view of men in general expressed by the mothers in the FGDs.

The majority of the fathers expressed a commitment to support their wives if they were HIV infected. It was seen by many fathers as a situation affecting them as a couple. Several of the fathers said that it would have been a motivation for them to test themselves:

The news would shock me and I would go and test too. If we are both infected, then we will follow the advice of the experts. If only she is infected, I will care for her till the end. (Father FGD)

### The acceptability of condoms

#### "A woman should not ask her husband to use condoms"

Condom use has been emphasized in the PMTCT programmes. Nevertheless, this was an issue arousing great controversy and reluctance. Preventing a child from infection did not seem to justify the use of condoms within marriage, according to most of the fathers. The fathers generally associated the use of condoms with unfaithfulness and thus as unacceptable within the marriage:

Why should I use a condom while I am her husband? (Father in-depth interview)

The general perception among fathers was that if the wife suggested that they should use condoms, either she suspected him of having been unfaithful or she had been unfaithful herself:

If she asks me to use condoms, questions are inevitable. First of all, what does she suspect? Why suddenly feel this way? (Father FGD)

Mothers were taught about the importance of condom use at the clinics and had a favourable view of them. However, several mothers would not ask their partner to use condoms owing to fear of their reactions:

A woman cannot protect herself; if she suggests using condoms it triggers a big fight in the house. (Mother FGD)

The mothers and fathers both stated that use of condoms was a decision for the husband:

It is not easy to tell me, the husband, to use a condom. I am the one who should ask. (Father FGD)

Safe sex and the use of condoms hence seemed to be the most sensitive and emotional component in the PMTCT programme. It interfered with established gender norms, and was defined primarily as an issue of trust between partners and not a measure to prevent the baby from getting HIV.

### The acceptability of infant feeding recommendations

#### Expectations to breastfeed

In the majority of cases (70.2%), infant feeding was a decision to be made by the mother. In the qualitative interviews, both fathers and mothers stated that the father did not get involved as long as the mother fed the infant according to the customary "mixed feeding" pattern. However, he would not accept exclusive replacement feeding or exclusive breastfeeding with early weaning without being given a reasonable explanation. In the wake of the HIV epidemic, he knew very well that not breastfeeding could be a sign of HIV infection in the mother:

I would demand to know why she does not breastfeed and suggest we go for HIV testing together. (Father in-depth interview)

Breastfeeding was seen as a maternal commitment and as a condition for infant survival. If a wife could not give a satisfactory explanation for not breastfeeding, the fathers said that she risked sanctions, such as being forced to breastfeed or even being divorced:

In our tradition if she refuses to breastfeed without a good reason, she would be endangering the marriage. (Father FGD)

Mothers stated that to be able to exclusively replacement feed or exclusively breastfeed with early weaning, an HIV-infected woman would have to disclose her HIV status to her partner:

You cannot decide to stop breastfeeding without telling him. You must tell him why you are doing it before stopping. (Mother FGD)

Although infant feeding was defined as the mother's responsibility, the findings pointed to a very clear interest in the way the infant was fed also on the part of the father. For a woman to be able to adhere to the PMTCT infant feeding recommendations, she needed the support of her partner.

## Discussion

This mixed-methods study on partner involvement in the PMTCT programme carried out in the Kilimanjaro region of Tanzania revealed that women's participation in the programme was highly appreciated by their partners, but that men's involvement was very limited. This was not primarily related to lack of knowledge and interest on the part of men, but seemed to be connected rather to the local definition of gender roles and responsibilities. The major obstacle was the definition and organization of the programme as fundamentally female oriented.

The following discussion will elaborate on the themes: (a) the antenatal clinic as a facilitator or a barrier for PMTCT programme implementation; (b) high partner disclosure rates as a sign of trust; and (c) how to involve the fathers in the PMTCT programme.

### The antenatal clinic: a facilitator or a barrier to PMTCT programme implementation

The expansion of HIV counselling and testing as part of the routine antenatal care services has increased the number of pregnant women being tested for HIV [19,20,38-41]. In this study, routine testing was highly acceptable among both men and women. The fear of partner disapproval about HIV testing that has been documented in other studies [4,5,33] was not found to be an obstacle in this study. The majority of the couples were aware that HIV testing was a component of the antenatal services, and they had discussed the issue before the woman attended.

A woman seeking voluntary counselling and testing, by contrast, was associated with distrust and was perceived as far less acceptable. Routine counselling and testing and the partner's positive view of the antenatal clinical activities seemed to have made it easier for a pregnant woman to be tested for HIV [20].

This study suggested a lower male testing rate at the antenatal clinic (3%) than other studies from eastern and southern Africa, which found testing rates ranging from 8% to 15% [7,9,18,20]. The qualitative data supported the numbers found in the PMTCT antenatal clinic registers. There was a discrepancy between the favourable attitude expressed by fathers to test for HIV during their spouses' pregnancy and the number of fathers who were actually tested at the antenatal clinics [17,18].

In this study, the close link between PMTCT services and the antenatal clinics appeared to have been a barrier to male involvement [17,42,43], indicating that the organization of the testing services rather than the attitude of fathers needs to be addressed. Attending the antenatal clinic was seen as "unmanly" to the extent that men feared being socially stigmatized if they accompanied their wives to the antenatal clinic [42-44]. It would be a signal of weakness and lack of masculinity and power. A man in this context is not supposed to follow his wife; he is supposed to take the lead. Several of the participants suggested that fathers could be offered testing in separate clinics or testing facilities distinct from the antenatal clinic in order to avoid the resistance connected to the antenatal clinic being seen as a female domain [33,45].

### The issue of trust

Almost all the mothers in both the quantitative and qualitative study who had been tested for HIV reported having told their partners their test results. However, the HIV status of individuals was not collected, and therefore the disclosure rates by HIV status cannot be given. Previous studies indicate that only half of the HIV-positive mothers disclose their results compared with HIV-negative mothers [8,10]. The disclosure pattern reported in this study seems to be higher than what has been reported in other studies, in which disclosure rates have ranged from 16% to 86% [14,15]. This could suggest bias due to perceived socially desirable answers, but it is also possible that the couples' prior awareness of and communication about HIV testing at the antenatal clinic could have facilitated disclosure [16].

Furthermore, the majority of the mothers reported during the quantitative and the qualitative interviews that their partners would have been their primary confidants if they were hypothetically HIV infected. Although the mothers feared their spouses' reactions to a positive test result, they expressed an obligation and a responsibility to tell them. These attitudes have been previously reported in the Kilimanjaro region [33].

Furthermore, it would be difficult for an HIV-infected mother to adhere to the infant feeding guidelines without disclosing her HIV status to her partner. The tendency of the mothers in the in-depth interviews to be more optimistic about their partners' potential reactions to a positive HIV test could be attributed to the in-depth interviews reflecting personal experiences, while the FGDs reflected community norms about men.

The fathers expressed a generally supportive attitude to a hypothetical HIV-infected spouse, contradicting the pessimistic view that was held among some of the mothers. This discrepancy between anticipated and actual consequences of HIV status disclosure has been demonstrated previously [8,10,14-16]. However, the views expressed by the fathers could reflect a socially desirable reaction to the hypothetical question. Fathers, who said that they would not have trusted their spouses in this hypothetical situation, admitted that they would have been likely to blame and even divorce them. Therefore, negative outcomes of HIV status disclosure are likely to exist and need to be acknowledged [15].

### Getting fathers involved

Gender hierarchy is an underestimated challenge in the PMTCT programme. The mother is expected to share the knowledge received at the clinic with her partner, and to ask him to be tested for HIV. In social and cultural context of the Kilimanjaro region however, it seemed to be socially unacceptable for a wife to tell her husband what to do or for a man to do what his wife asked him to do. Couple counselling and testing at the antenatal clinic would have been an ideal option [7,9,18], but the husbands demonstrated reluctance to attend the "female" antenatal clinic when asked to do so by their wives.

The low acceptance of couple counselling and testing found in this study is consistent with research in other settings in sub-Saharan Africa [7,18,46]. Therefore, it could be argued that the first step should be to encourage the fathers to undergo tests. This could be more readily achievable if the fathers were directly invited to be tested by health personnel, for example, by giving them invitation letters, as has been suggested by Theuring *et al *[17]. In this way, men may feel more included in the PMTCT programme and therefore more likely to act upon it and take responsibility.

Research is required to explore alternative organization for partner testing within the PMTCT programme. Options could include facilities for men only or facilities designed specifically for pregnant couples. Further, community mobilization is necessary to increase male participation in PMTCT. A national HIV-testing campaign was launched by the President of Tanzania in July 2007, and by the end of December 2007, 3.2 million people had been tested [21]. It remains a research question if similar strategies could be used to promote male partner testing when the spouse is pregnant.

Similarly, it was the women who were encouraged to ask their partners to use condoms. However, traditional gender roles make it very difficult for a woman to ask her husband to use condoms without compromising his authority and their trust relation. Hence, messages about condom use should be communicated to the man directly. In general, however, the promotion of condom use in marriage is likely to be difficult since it is seen to imply distrust [10]. Nevertheless, conjugal condom use has been accepted when both partners have been through HIV counselling and testing [8], further substantiating the importance of partner testing.

### Methodological limitations

The concurrent mixed-methods design did not allow for information gained by one method to influence the next method, which would had been the case if a sequential design had been conducted.

#### Truth value

The principal investigator was not conversant in the local languages. An interpreter was used when the in-depth interviews were conducted by the principal investigator. This might have created a distance between the interviewer and the interviewee. The FGDs were conducted by an experienced moderator trained in discussing sensitive topics. The principal investigator had a continuous discussion regarding responses and decided on the need for additional and supplemental information. Each FGD was transcribed and translated before the next FGD was performed so that these adjustments could be made if necessary. The principal investigator analyzed qualitative data from transcripts that had been translated to English, which diluted the richness of the data. Furthermore, translation is always associated with various aspects of meaning loss; therefore, some of the original meanings might be lost.

Convenience sampling, selecting those most readily available, has the lowest credibility of the different qualitative sampling strategies [47]. In addition, familiarity with the main research assistant or the village leader may have affected the respondents' willingness to participate. In some of the focus groups, several of the participants were familiar with each other, which may have inhibited openness when discussing sensitive issues.

A limitation of the quantitative study was the facility-based design, which could have made it difficult for the mother to decline participation in the study and could have introduced a social desirability bias. It would also have been preferable to use male reports when studying male involvement in PMTCT, but female reports have been found to be an acceptable alternative [18].

#### Applicability

Subsequent sampling of the mothers in the selected clinics may inhibit generalization of the quantitative findings, as opposed to a randomized study. However, the Moshi District is known for its high clinical attendance, so it is likely that the sample is representative of Moshi pregnant women. But the area is known for a high educational level and high utilization of health services [27,28,48], so it might not be representative of the country as a whole.

## Conclusions

Women's participation in the PMTCT programme is highly accepted in this region. However, the organization of the programme is a barrier to male attendance. Men tend to define PMTCT as a woman's responsibility. Women do not have the authority to request their husbands to test for HIV, and the arena for testing, the antenatal clinic, is defined as a typical female domain, where men are out of place.

Empowering women remains a huge challenge. Empowering men to participate by creating a space within the PMTCT programme that is male friendly should be feasible and should be highly prioritized for the PMTCT programme to achieve its potential.

The PMTCT programme in sub-Saharan Africa has already suffered a defeat owing to the gap between global politics and local realities, namely the lack of concern for the local context of infant feeding. Similarly, the lack of concern for customary gender roles and acceptable arenas for male participation may prevent efforts of male involvement in the PMTCT programme from succeeding in sub-Saharan Africa in the near future. Further research addressing cultural issues is required to explore alternative organization of the PMTCT programme to facilitate male participation.

## Competing interests

The authors declare that they have no competing interests.

## Authors' contributions

All authors participated in the design and planning of the study. The field work was mainly conducted by EFF; the analysis and write up was carried out mainly by EFF, KMM, TT, MMdP, SEM and IMSE. All authors read and approved the final manuscript.

## Authors' information

EFF is a medical doctor and PhD candidate. She has research experience from a qualitative infant feeding study in Zambia. KMM is associated professor at the Department of Nursing at Bergen University College. She is a nurse and a social scientist with a PhD in maternal and reproductive health in the Kilimanjaro region. She has extensive experience in qualitative methods in research in Tanzania and Ethiopia. TT has a Master in African Linguistics and is a paediatrician and professor at the Centre for International Health at University of Bergen, with extensive experience from in health-related research in sub-Saharan Africa. MMdP is a nutritionist with a PhD in public health nutrition. She has extensive experience in mixed-methods research in Tanzania, South Africa and India. SEM is a medical doctor from Tanzania, working at a hospital in Moshi. She has a PhD in public health in the Kilimanjaro region. IMSE is a medical doctor with a PhD in child health and nutrition and has experience in mixed-methods research in Uganda.
